# Patient, kidney, and pancreas survival in pancreas after kidney transplantation *versus* simultaneous pancreas and kidney transplantation: meta-analysis

**DOI:** 10.1093/bjsopen/zrac108

**Published:** 2022-09-09

**Authors:** Wenrui Xue, Zhen Huang, Yu Zhang, Xiaopeng Hu

**Affiliations:** Department of Urology, Beijing Youan Hospital of Capital Medical University, Beijing, China; Department of Urology, Beijing Youan Hospital of Capital Medical University, Beijing, China; Department of Urology, Beijing Youan Hospital of Capital Medical University, Beijing, China; Department of Urology, Beijing Chaoyang Hospital of Capital Medical University, Beijing, China

*Dear Editor*

Both pancreas after kidney transplant (PAK) and simultaneous pancreas and kidney transplant (SPK) are treatment options for patients with type 1 diabetes (T1DM) and end-stage kidney disease (ESKD), but they have different risks and benefits. Presently, SPK is more widespread than PAK because SPK requires only one operation^1^. PAK is a recommended option for patients with living kidney donors to avoid long-term uraemia while waiting for cadaveric pancreas transplantation and to increase the survival of allograft kidney transplantation^2^. The aim of this study was to compare PAK and SPK in terms of patient, kidney, and pancreas survival.

Online databases were used to locate studies of patient with T1DM and ESKD undergoing PAK and SPK up to 20 January 2022 (*Supplementary material* and *Fig. S1*). A total of 47 986 patients were identified from 24 studies, including 9093 patients undergoing PAK and 38 893 patients undergoing SPK (*Table S1)*. Patient, kidney, pancreas survival rate as well as human leucocyte antigens (HLA) mismatch rate in patients undergoing PAK *versus* SPK at 1, 3, 5, and 10 years in the included studies are shown in *Table 1*. Quality ranged on the Newcastle–Ottawa Scale from six to nine (*Table S2)*. The definitions of pancreas and kidney rejection, the rejection ratios of PAK and SPK, and the details of the rejection treatment used in all the included articles are given in *Table S3*.

**Table 1:** Patient, kidney, and pancreas survival rate and human leucocyte antigens mismatch rate of patients undergoing pancreas after kidney transplant *versus* simultaneous pancreas and kidney transplant at 1, 3, 5, and 10 years in the included studies

First author	Total number of recipients		Patient survival rate for the following years	Kidney survival rate for the following years	Pancreas survival rate for the following years	HLA mismatch rate
	PAK	SPK				
Abhinav Humar s1	205	193	1(96 *versus* 91):3(90 *versus* 90)	1(100 *versus* 85):3(93.6 *versus* 83.6):5(90 *versus* 76)	1(77.9 *versus* 78):3(61.7 *versus* 74.1)	1.3 *versus* 1.6
Ayhan Dinckan s2	34	21	5(97 *versus* 95.2)	5(91.2 *versus* 95.2)	1(79.4 *versus* 85.7):3(70.6 *versus* 71.4):5(61.8 *versus* 61.9)	8.8 *versus* 14.3
B.L.Kasiske s3	1230	4316	1(95.5 *versus* 96)	NA	1(82 *versus* 88)	NA
Bor-Uei Shyr s4	24	37	1(96 *versus* 97):3(96 *versus* 97):5(96 *versus* 95):10(96 *versus* 95)	NA	1(100 *versus* 100):3(100 *versus* 100):5(100 *versus* 97):10(100 *versus* 91)	12.5 *versus* 8
Edmund Huang s5	389	1402	1(100 *versus* 98):3(96 *versus* 95):5(89 *versus* 91)	1(100 *versus* 95):3(95 *versus* 90):5(83 *versus* 80)	1(81.7 *versus* 86):3(70 *versus* 81):5(53 *versus* 74)	8.5 *versus* 2.4
Farney s6	17	19	1(70 *versus* 84)	1(100 *versus* 89)	1(100 *versus* 95)	17 *versus* 19.5
Fridell JA s7	61	142	1(98 *versus* 95):3(90 *versus* 90)	1(98 *versus* 96):3(92 *versus* 90)	1(95 *versus* 90):3(88 *versus* 85)	6.6 *versus* 2.8
Jeffrey M. Venstrom s8	838	5379	1(95 *versus* 95):3(84.5 *versus* 87.5)	NA	NA	NA
Jens Gunther Brockmann s9	17	10	1(100 *versus* 100):3(100 *versus* 100)	1(88 *versus* 100)	1(88 *versus* 100)	NA
Jonathan A. Fridell s10	3358	12308	1(94 *versus* 93.7):3(88 *versus* 89):5(83 *versus* 86.4):10(63.2 *versus* 70)	1(93.7 *versus* 85.5):3(93.7 *versus* 85.5):5(88 *versus* 78):10(66 *versus* 61)	1(84.4 *versus* 87.5):3(69 *versus* 81.2):5(62.5 *versus* 75):10(44.4 *versus* 58.7)	14.1 *versus* 14.1
Muthusamy s11	15	83	NA	NA	1(80 *versus* 90):3(60 *versus* 88)	NA
M.R. Laftavi s12	23	25	1(96 *versus* 100):3(87 *versus* 88):5(87 *versus* 88):10(87 *versus* 88)	1(96 *versus* 96):3(87 *versus* 96):5(83 *versus* 96):10(83 *versus* 84)	1(96 *versus* 96):3(87 *versus* 96):5(83 *versus* 96):10(83 *versus* 84)	17.4 *versus* 20
M. Durlik s13	9	159	1(88.9 *versus* 86.2):3(88.9 *versus* 83):5(88.9 *versus* 83):10(88.9 *versus* 83)	1(100 *versus* 87):3(100 *versus* 85):5(100 *versus* 85):10(100 *versus* 80)	1(100 *versus* 71):3(100 *versus* 67):5(100 *versus* 63):10(100 *versus* 60)	NA
Nedo Poommipanit s14	807	5580	1(95 *versus* 94):3(92 *versus* 90):5(86 *versus* 86)	1(98 *versus* 93):3(94 *versus* 85):5(85 *versus* 77)	1(81 *versus* 86):3(65 *versus* 78):5(55 *versus* 73)	0.45 *versus* 0.08
Perosa M. s15	94	254	1(92.5 *versus* 81.8):3(91.5 *versus* 78):5(90.4 *versus* 75.2)	NA	1(86 *versus* 70):3(73.4 *versus* 68):5(65 *versus* 60)	NA
Pedro Ventura-Aguiar s16	18	139	1(100 *versus* 98):3(83 *versus* 95):5(77.8 *versus* 92):10(77.8 *versus* 90)	1(100 *versus* 98):3(100 *versus* 95):5(100 *versus* 90):10(100 *versus* 88)	1(83.3 *versus* 96):3(72 *versus* 90):5(61 *versus* 82):10(27.8 *versus* 80)	16.1 *versus* 2.2
Rainer WG s17	1714	6995	1(95 *versus* 95):3(88 *versus* 90)	NA	NA	NA
Robert Ollinger s18	47	442	NA	NA	1(70 *versus* 82):3(66 *versus* 80):5(62 *versus* 69)	NA
R.J. Stratta s19	35	162	5(86 *versus* 86)	5(80 *versus* 74)	5(66 *versus* 65)	7.7 *versus* 2.8
Sandesh Parajuli s20	24	611	NA	1(100 *versus* 95):3(96 *versus* 90):5(96 *versus* 85):10(96 *versus* 75)	31(100 *versus* 89):3(83 *versus* 85):5(83 *versus* 80):10(83 *versus* 70)	11.3 *versus* 0.7
Timothy S. Larson s21	47	25	1(93.6 *versus* 100):3(85 *versus* 100):5(81 *versus* 92)	NA	1(87 *versus* 92):3(81 *versus* 92):5(81 *versus* 80)	NA
Tadahiiro Uemura s22	5	17	1(100 *versus* 100):3(100 *versus* 100)	1(100 *versus* 100):3(100 *versus* 100)	1(100 *versus* 100):3(100 *versus* 100)	NA
T. Ito s23	39	232	5(97 *versus* 88)	NA	1(87 *versus* 87.5):3(64 *versus* 86):5(48.7 *versus* 82.8)	NA
Tomimaru s24	48	344	NA	NA	1(85 *versus* 87):3(66.7 *versus* 85.5):5(52 *versus* 83):10(42 *versus* 74.7)	NA

Values are *n* (PAK:SPK). HLA, human leucocyte antigen; PAK, pancreas after kidney transplant; SPK, simultaneous pancreas and kidney transplant; NA, not available. References to the studies can be found in Supplementary material.

Patients with PAK had a significantly higher 1-year patient survival than patients with SPK (OR 1.11, 95 per cent c.i. 1.00 to 1.24), whereas patients with PAK had a lower 10-year patient survival than patients with SPK (OR 0.73, 95 per cent c.i. 0.67 to 0.79) (*Fig. S2*). Patients with PAK had a higher 1-year (OR 5.72, 95 per cent c.i. 4.38 to 7.48), 3-year (OR 2.51, 95 per cent c.i. 2.21 to 2.84), 5-year (OR 1.74, 95 per cent c.i. 1.37 to 2.21), and 10-year (OR 1.25, 95 per cent c.i. 1.16 to 1.36) kidney graft survival than after SPK (*Fig. S3*), but lower 1-year (OR 0.79, 95 per cent c.i. 0.66 to 0.94), 3-year (OR 0.55, 95 per cent c.i. 0.46–0.65), and 5-year (OR 0.55, 95 per cent c.i. 0.44 to 0.69) pancreas graft survival than after SPK (*Fig. S4*). The incidence of pancreas rejection (OR 2.37, 95 per cent c.i. 1.47 to 3.85) and the HLA mismatch rate (OR 2.22, 95 per cent c.i. 1.19 to 4.15) were higher in patients with PAK than with SPK (*Figs. S5* and *S6*).

Patients with SPK have better long-term survival than patients with PAK, which may indicate that rejection is difficult to detect without simultaneous kidney transplantation^3^. Kidney graft survival in PAK is significantly better, in part due to selection bias, as only recipients with good renal function after kidney transplantation will proceed to pancreas transplantation. Another main reason for higher kidney graft survival in the PAK group was that the proportion of living donors was significantly higher. The pancreas graft long-term survival rate in PAK is still slightly lower than that of SPK, and an adverse pancreas outcome after PAK may be due to technical complications and immunological issues^4^. Higher graft survival yields lower mortality and preventing both immune and non-immune causes of graft failure is critical in reducing post-transplant mortality^5^.

This study has some limitations, the studies included a large range of years, and earlier transplantation technology had a certain impact on the patient survival and graft survival of the two groups (SPK *versus* PAK recipients had a higher incidence of kidney graft loss due to technical reasons in 2000: 2.1 per cent *versus* 0 per cent . At present, the technical failure rate of SPK as a cause of allograft failure is similar to that of PAK). Furthermore, limited to the results in the included literature, the effects of other post-transplant complications, such as thrombosis, infection, obesity, smoking, and coronary heart disease, were not taken into account.

## Supplementary Material

zrac108_Supplementary_Data

## Data Availability

The data underlying this article are available from the corresponding authors upon request.
