# Supplementary material for: Patient, kidney, and pancreas survival in pancreas after kidney transplantation versus simultaneous pancreas and kidney transplantation: meta-analysis
Source: BJS Open. 2022 Sep 9;6(5):zrac108. doi: 10.1093/bjsopen/zrac108 (PMC9459350; doi:10.1093/bjsopen/zrac108)
Supplement: zrac108_Supplementary_Data [file zrac108_supplementary_data.docx]

**Title**

Patient, Kidney, and Pancreas Survival in Pancreas after Kidney Transplantation versus Simultaneous Pancreas and Kidney Transplantation: Meta-analysis

**Authors**

Wenrui Xue^1^, Zhen Huang^1^, Yu Zhang^1^, Xiaopeng Hu^2^

**^1^Affiliation：**Beijing Youan Hospital of Capital Medical University.

**^2^Affiliation：**Beijing Chaoyang Hospital of Capital Medical University.

**Corresponding author.**

**Xiaopeng Hu** and **Yu Zhang** are the co-corresponding authors

Xiaopeng Hu, Department of Urology, Beijing Chaoyang Hospital, No 8 of Gongti South Street, Beijing, China. 100020. Email address: [selihebe2019@163.com](mailto:selihebe2019@163.com)

Yu Zhang, Department of Urology, Beijing Youan Hospital, No 8 of Xitou Tiao, Beijing, China. 100069. Email: [wenruixue@hotmail.com](mailto:wenruixue@hotmail.com、)

**Supplementary Materials - Index**

| **Supplementary Methods** |  |
| --- | --- |
| Initial Search | *pag. 2* |
| Study Selection and Analysis Risk of Bias in Individual StudiesData Synthesis |  |
| **Supplementary Figures and Tables** |  |
| Figure S1-S6, Table S1-S3 | *pag. 3-13* |
|  |  |
| **References** | *pag. 14-15* |
|  |  |

**Supplementary Methods**

**MATERIALS AND METHODS**

**Initial Search**

The search strategy followed the recommendations in the Cochrane Handbook and was reported according to the PROSPERO protocol and the PRISMA statement.

Online databases, including PubMed, Cochrane, Embase Ovid, and MEDLINE Ovid, were used to locate the studies of T1DM patients with ESKD undergoing PAK and SPK up to January 20, 2022. The included studies compared the outcome data of patients or patients with grafts who were followed up for at least 1 year or more after PAK or SPK treatment. Exclusion criteria included studies with outcome data in the form of case reports, comments, letters and conference papers, studies on pediatric organ transplantation, studies where data could not be extracted and postoperative follow-up time was not clear, and studies involving patients on transplant waiting lists. Full-text articles were reviewed by two independent reviewers, and any discrepancies were resolved through discussion.

We used the following keywords to search: "PAK" or "Pancreas after kidney transplantation" combined with "SPK" or "Simultaneous pancreas and kidney transplantation". The search was limited to English but included studies conducted outside the United States. To reduce the publication bias of our systematic analysis, we were not restricted by publication status or any other research characteristics, and references to articles that met the criteria were also checked in the literature search.

**Study Selection and Analysis**

The search strategy generated initial article titles and abstracts, and then two review members independently screened eligible studies. For each study, the data collected included date of publication, country, first author, start and end of study data collection, number, age (year), gender, follow-up time, immunosuppression protocol, HLA mismatch, rejection and treatment. For articles with different first authors from the same center, we included the articles with the most recent and complete data. The full texts of the trials that met the criteria were investigated. The primary predetermined outcomes were patient survival, kidney graft survival, and pancreas graft survival at 1, 3, 5, and 10 years after transplantation. Secondary outcomes were graft rejection and HLA mismatch rates. The inclusion criteria were articles comparing patient survival or graft survival over 1 year after PAK and SPK. The exclusion criteria were as follows: ① Articles in which valid patient or graft survival data could not be extracted although PAK and SPK were compared; ② articles that compared patients on the waiting list rather than patients after transplantation; ③ articles on the transplantation of children; and (4) articles without clear data about the years after transplantation.

## Risk of Bias in Individual Studies

The Newcastle-Ottawa Scale (NOS) recommended by the Cochrane Handbook was adopted for quality assessment. The NOS contains 8 items within 3 domains, and the total maximum score is 9. A score less than 6 points indicates that the research quality is decreased. Disagreements between reviewers regarding the risk of bias were resolved by discussion with the third reviewer.

## Data Synthesis

We used RevMan 5.5 software to conduct a systematic analysis of the data results. A funnel plot was used to test publication bias. Statistical heterogeneity was assessed using I^2^ statistics. If P > 0.05 or I^2^＜50% in the heterogeneity test, the fixed effects model was used; if P < 0.05 or I^2^ > 50% in the heterogeneity test, the random effects model was used.

**Supplementary Figures and Tables**

**Figure S1. Study selection**


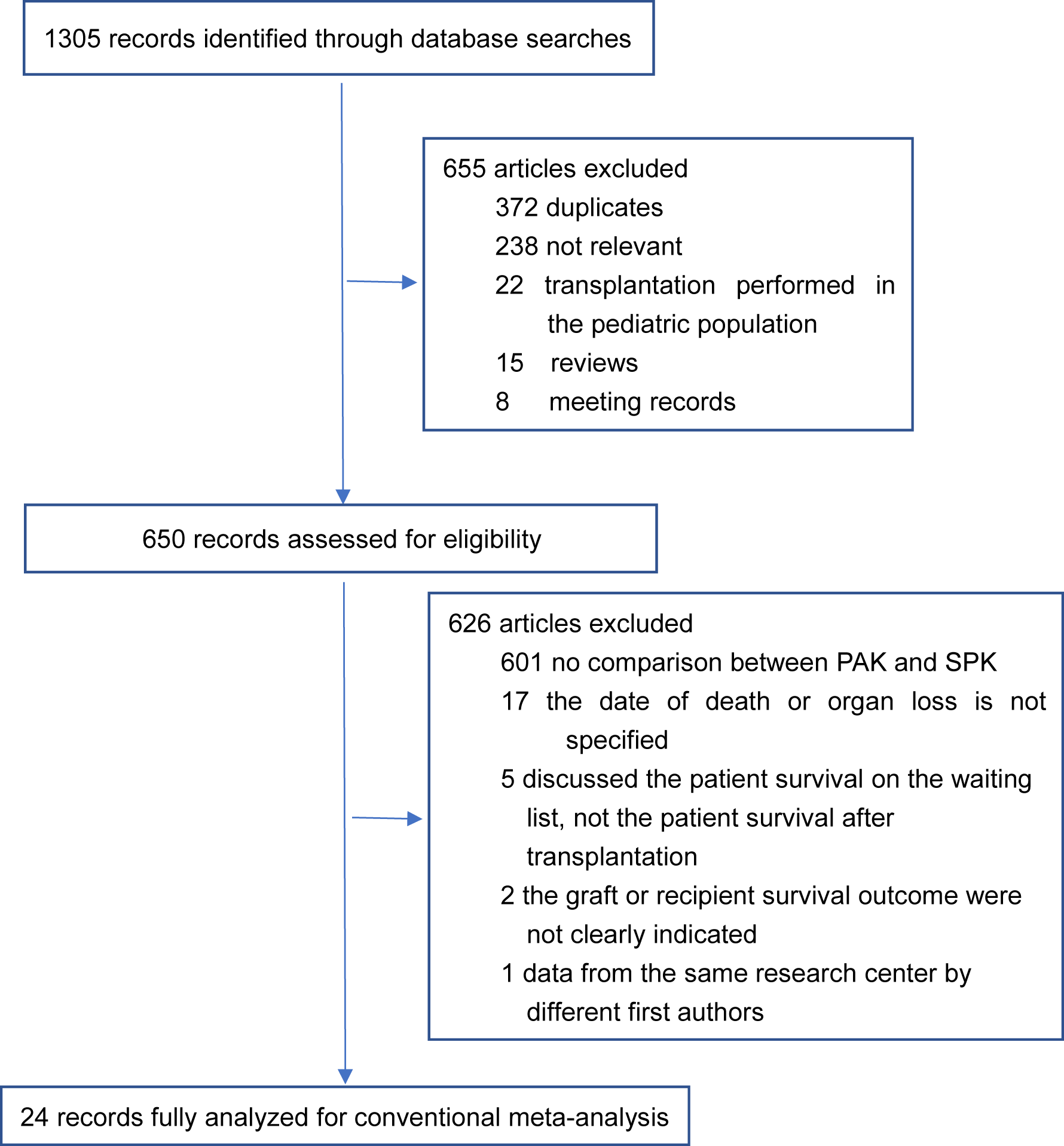


**Figure S2. Patient survival of PAK versus SPK at 1 year(A), 3 years(B), 5 years(C), and 10 years(D).**


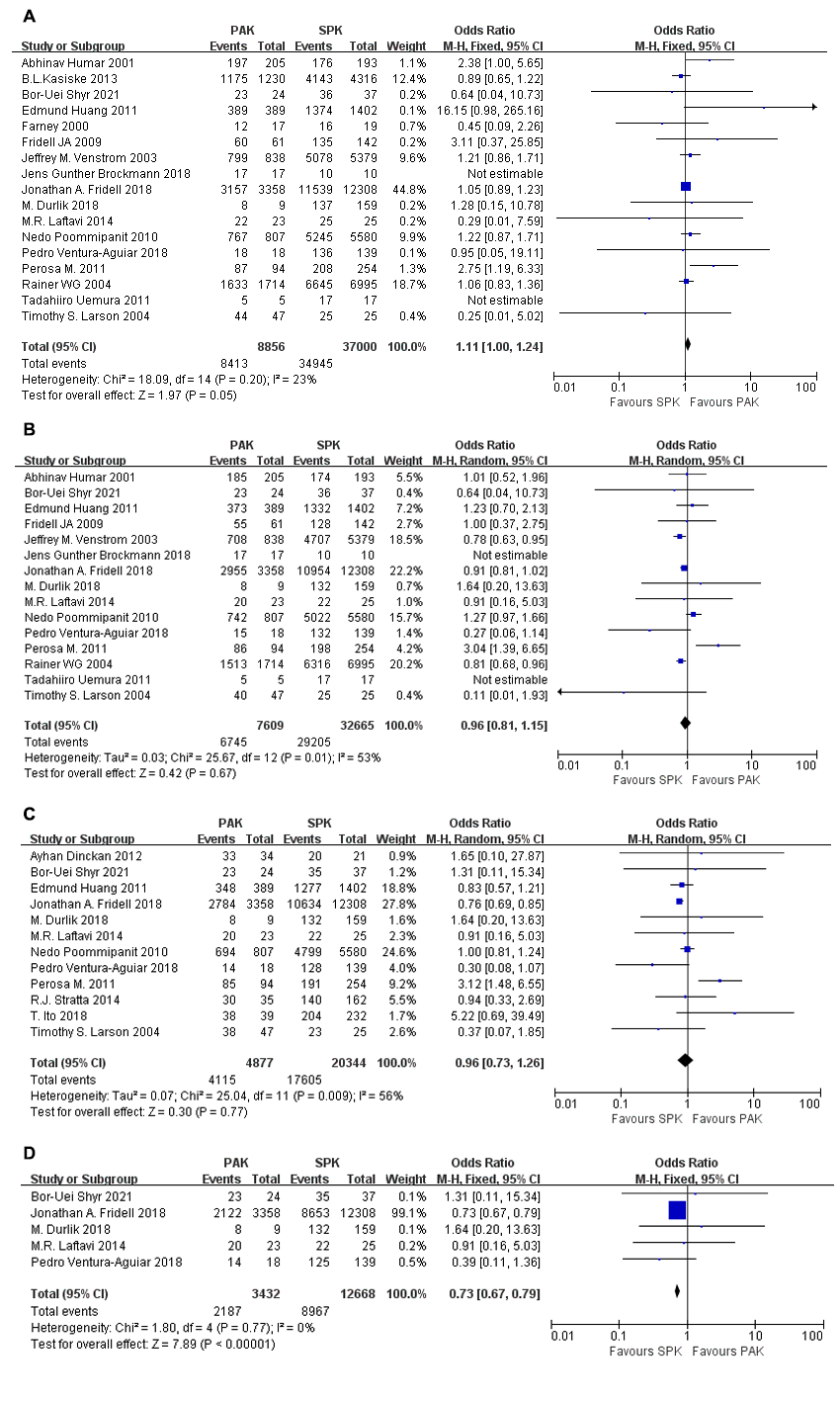


**Figure S3. Kidney graft survival of PAK versus SPK at 1 year(A), 3 years(B), 5 years(C), and 10 years(D).**


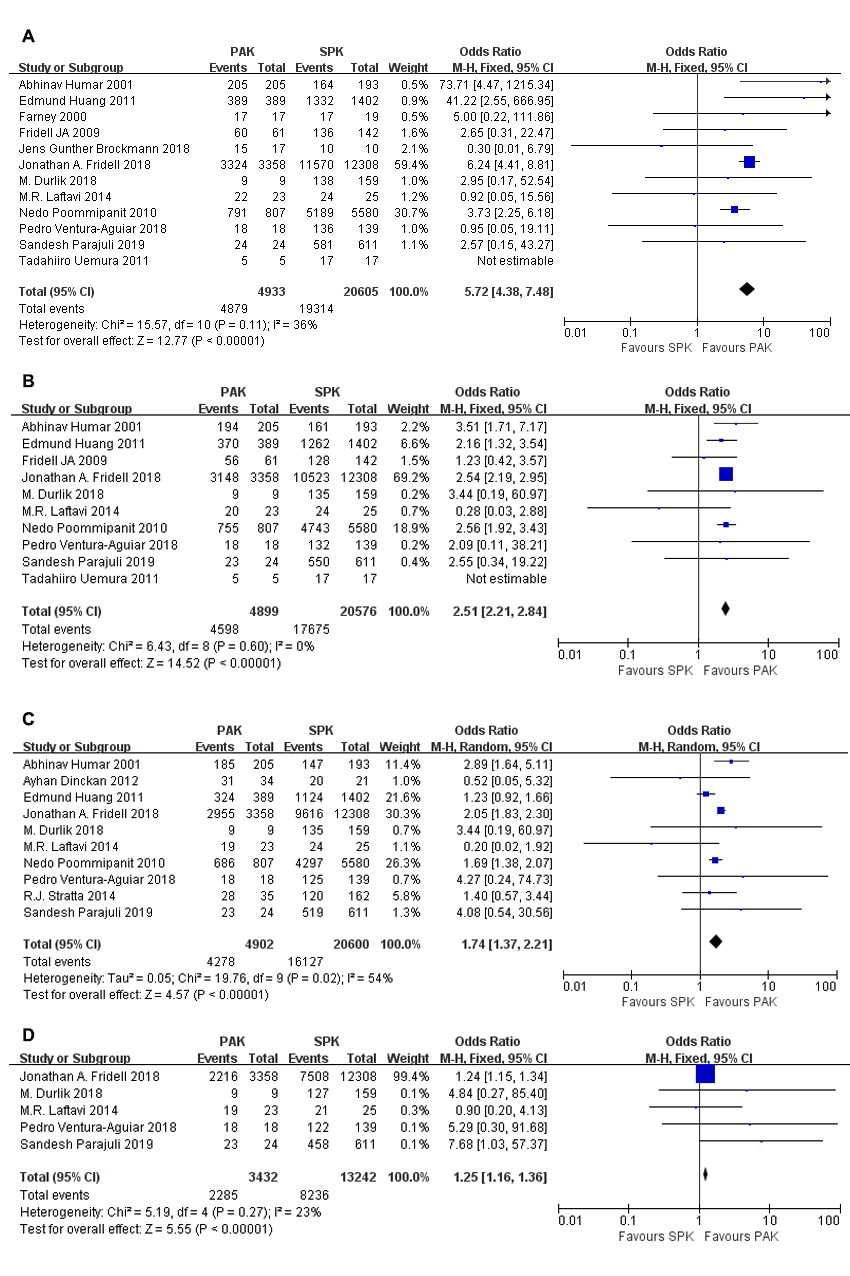


**Figure S4. Pancreas graft survival of PAK versus SPK at 1 year(A), 3 years(B), 5 years(C), and 10 years(D).**


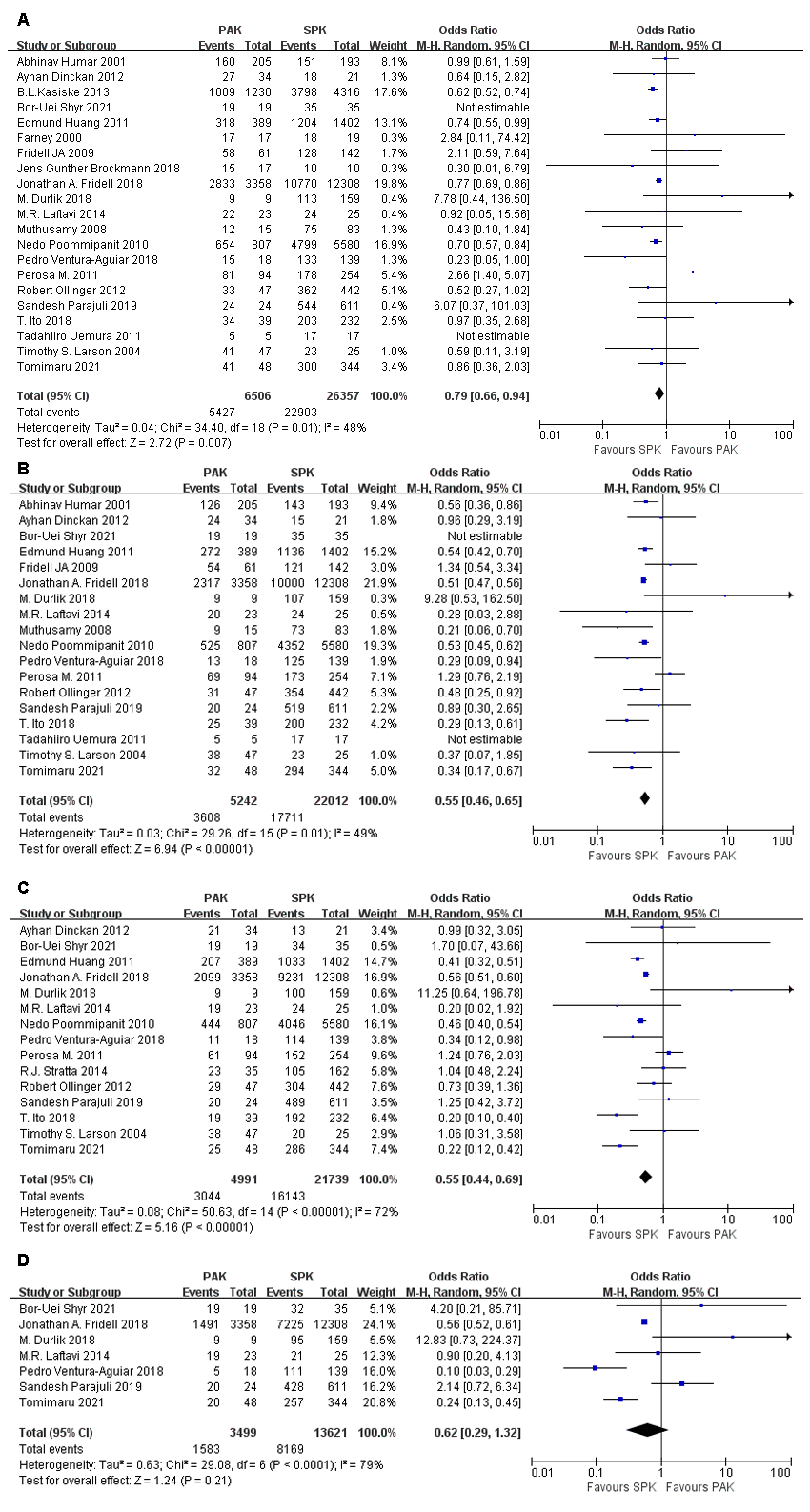


**Figure S5. Pancreas(A) and kidney(B) rejection of PAK versus SPK.**


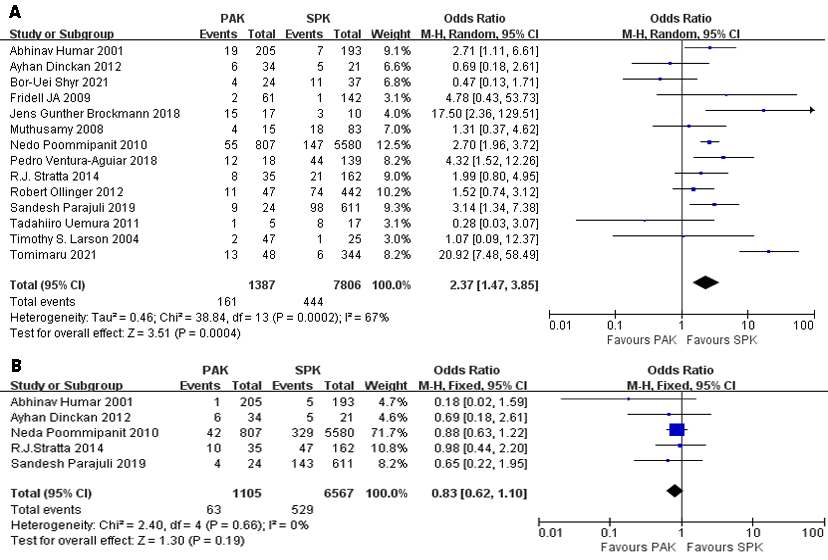


**Figure S6. HLA mismatch of PAK versus SPK.**





**Table S1.**

| **Table S1. Baseline Demographics of Relevant Randomized Clinical Trials** | | | | | | | | | | | | | | |
| --- | --- | --- | --- | --- | --- | --- | --- | --- | --- | --- | --- | --- | --- | --- |
| First author, year | Country | Data sources | Study period | PAK | | | SPK | | | Follow-up (yr) | | | Immunosuppressive therapy | Mean HLA mismatches(n): PAK vs SPK |
|  |  |  |  | Patients | Median/Mean age (yr) | Sex(F/M) | Patients | Median/Mean age (yr) | Sex(F/M) | Patient Survival | Kidney Survival | Pancreas Survival |  |  |
| Abhinav Humar 2001 | USA | University of Minnesota | 1994-2000 | 205 | 37.3 | 81/112 | 193 | 40.2 | 88/117 | 1,3 | 1,3,5 | 1,3 | Polyclonal antilymphocyte agent + tacrolimus+ MMF | 2.6 vs 3.1 |
| Ayhan Dinckan 2012 | Turkey | Akdeniz University Organ Transplantation Institution | 2003-2010 | 34 | 32.0±6.0 | 9/25 | 21 | 33.6±6.8 | 8/13 | 5 | 5 | 1,3,5 | ATG+ tacrolimus/cyclosporine+ MMF+ methylprednisolone | 3.2 vs 3.2 |
| B.L.Kasiske 2013 | USA | The Scientific Registry of Transplant Recipients (SRTR) | 2005-2010 | 1230 | 43.3(37-49) | 394/836 | 4316 | 42.5(36-49) | NA | 1 | NA | 1 | Not mention | NA |
| Bor-Uei Shyr 2021 | Taiwan | Taipei Veterans General Hospital | 2003-2020 | 24 | 38(20-55) | 13/11 | 37 | 37(26-58) | 16/21 | 1,3,5,10 | NA | 1,3,5,10 | Basiliximab/ATG+ tacrolimus + MMF+ prednisolone | 3 vs 3 |
| Edmund Huang 2011 | USA | Organ Procurement and Transplantation Network | 2000-2010 | 389 | 41(34-47) | 172/217 | 1402 | 42(36-48) | 644/758 | 1,3,5 | 1,3,5 | 1,3,5 | Tacrolimus- and mycophenolate- based immunosuppression | 33 vs 33 |
| Farney 2000 | USA | University of Maryland School of Medicine | 1998-1999 | 17 | 43±8 | 4/13 | 19 | 38±6 | 6/13 | 1 | 1 | 1 | Tacrolimus+ MMF + prednisone, received 10 days of OKT3 | 2.9 vs 3.7 |
| Fridell JA 2009 | USA | Indiana University | 2003-2007 | 61 | 46 | 27/34 | 142 | 41 | 46/96 | 1,3 | 1,3 | 1,3 | ATG+ tacrolimus+ sirolimus/MMF | 4.1 vs 4.3 |
| Jeffrey M. Venstrom 2003 | USA | Organ Procurement and Transplantation Network | 1995-2000 | 838 | NA | NA | 5379 | NA | NA | 1,3 | NA | NA | Not mention | NA |
| Jens Gunther Brockmann 2018 | Kingdom of Saudi Arabia | King Faisal Specialist Hospital and Research Center | 2014-2016 | 17 | 33(22.5-52.8) | 9/8 | 10 | 33(22.5-52.8) | 2/8 | 1,3 | 1 | 1 | Lymphocyte depleting antibody+ thymoglobulin/alemtuzumab+ Tacrolimus+ MMF+ methylprednisolone | NA |
| Jonathan A. Fridell 2018 | USA | Organ Procurement and Transplantation Network | 1995-2010 | 3358 | 42 | 1431/1927 | 12308 | 40 | 5022/7286 | 1,3,5,10 | 1,3,5,10 | 1,3,5,10 | Not mention | 475 vs 1733 |
| Muthusamy 2008 | UK | Churchill Hospital | 2004-2007 | 15 | 42±7.6 | 6/9 | 83 | 42±7.6 | 32/51 | NA | NA | 1,3 | Alemtuzumab+ tacrolimus +MMF | NA |
| M.R. Laftavi 2014 | USA | University of Arizona | 2001-2013 | 23 | 41±7 | 6/17 | 25 | 45±6 | 8/17 | 1,3,5,10 | 1,3,5,10 | 1,3,5,10 | ATG+ tacrolimus+ MMF+ steroids. | 3.9 vs 4.5 |
| M. Durlik 2018 | Poland | Central Clinical Hospital | 2004-2017 | 9 | 38±8 | NA | 159 | 38±8 | NA | 1,3,5,10 | 1,3,5,10 | 1,3,5,10 | ATG+ MMF+ tacrolimus+ prednisone | NA |
| Nedo Poommipanit 2010 | USA | Organ Procurement and Transplantation Network | 2000-2007 | 807 | 38.85±7.64 | 353/454 | 5580 | 40.17±7.91 | 2174/3406 | 1,3,5 | 1,3,5 | 1,3,5 | Not mention | 3.6 vs 4.4 |
| Perosa M. 2011 | BraziL | Sa˜o Camilo Hospitals | 1996-2009 | 94 | NA | NA | 254 | 37.4(21-65) | 115/139 | 1,3,5 | NA | 1,3,5 | ATG**+** Tacrolimus+ MMF+ steroids | NA |
| Pedro Ventura-Aguiar 2018 | Spain | Hospital Clinic, Barcelona | 2007-2015 | 18 | 39.5±6 | 8/10 | 139 | 41.6±7.1 | 51/88 | 1,3,5,10 | 1,3,5,10 | 1,3,5,10 | ATG+ tacrolimus+ MMF+ steroids-methylprednisolone | 2.9 vs 3.1 |
| Rainer WG 2004 | USA | Organ Procurement and Transplantation Network | 1995-2003 | 1714 | 40.5±7.7 | 732/982 | 6995 | 39.1±8.1 | 2987/4008 | 1,3 | NA | NA | Not mention | NA |
| Robert Ollinger 2012 | Austria. | University Hospital Innsbruck | 1979-2011 | 47 | 41.6±9.4 | NA | 442 | 41.6±9.4 | NA | NA | NA | 1,3,5 | CyA+ azathioprine/MMF**+** steroids | NA |
| R.J. Stratta 2014 | USA | Wake Forest School of Medicine | 2001-2013 | 35 | 42.2±8.7 | 18/17 | 162 | 42.7±11.3 | 68/94 | 5 | 5 | 5 | ATG/alemtuzumab + tacrolimus+ MMF+ corticosteroids/steroid | 2.7 vs 4.5 |
| Sandesh Parajuli 2019 | USA | University of Wisconsin School  of Medicine and Public Health | 2000-2016 | 24 | 45.9±1.7 | 11/13 | 611 | 41.3±0.3 | 239/372 | NA | 1,3,5,10 | 1,3,5,10 | ATG/alemtuzumab/basiliximab+ tacrolimus+ MMF | 2.7 vs 4.4 |
| Timothy S. Larson 2004 | USA | Mayo  Clinic, Rochester | 1998-2002 | 47 | 42.6±7.2 | 21/26 | 25 | 42.8±7.6 | 10/15 | 1,3,5 | NA | 1,3,5 | ATG+ tacrolimus+ MMF+ prednisone | NA |
| Tadahiiro Uemura 2011 | USA | The Pennsylvania  State University | 2006-2010 | 5 | 41.0(28-57) | NA | 17 | 41.0(28-57) | NA | 1,3 | 1,3 | 1,3 | Alemtuzumab+ tacrolimus+ MMF+ steroid-free | NA |
| T. Ito 2018 | Japan | Japan Society for  Pancreas and Islet Transplantation | 2004-2016 | 39 | NA | NA | 232 | NA | NA | 5 | NA | 1,3,5 | Not mention | NA |
| Tomimaru 2021 | Japan | Japan Society for Pancreas and Islet Transplantation | 2000-2019 | 48 | 44(24-69) | NA | 344 | 44(24-69) | NA | NA | NA | 1,3,5,10 | ATG**+** basiliximab**+** Tacrolimus/CyA+ MMF+ steroids | NA |

Abbreviation: MMF, mycophenolate mofetil; ATG, Anti-thymocyte globulin; OKT3, OKT3 Monoclonal Antibody; CyA, cycolosporin; HLA, human leukocyte antigen; NA, Not Applicable.

**Table S2.**

| **Table S2. Results of quality assessment using the Newcastle-Ottawa Scale for case-control studies** | | | | | | | | | |
| --- | --- | --- | --- | --- | --- | --- | --- | --- | --- |
| Study | Selection | | | | Comparability | Outcome | | | Quality score |
|  | Representativeness of the exposed cohort | Selection of the non-exposed cohort | Ascertainment of exposure | Demonstration that outcome of interest was not present at start of study | Comparability of cohorts on the basis of the design or analysis | Ascertainment of outcome | Was follow-up long enough for outcomes to occur | Adequacy of follow up of cohorts |  |
| Abhinav Humar 2001 | ★ | ★ | ★ | ★ | ★★ | ★ | ★ | ★ | 9 |
| Ayhan Dinckan 2012 | ★ | ★ | ★ | ★ | ★★ | ★ | ★ | ★ | 9 |
| B.L.Kasiske 2013 | ★ | ★ | ★ | ★ | ★ | ★ |  |  | 6 |
| Bor-Uei Shyr 2021 | ★ | ★ | ★ | ★ | ★★ | ★ | ★ | ★ | 9 |
| Edmund Huang 2011 | ★ | ★ | ★ | ★ | ★ | ★ | ★ | ★ | 8 |
| Farney 2000 | ★ | ★ | ★ | ★ | ★ | ★ |  | ★ | 7 |
| Fridell JA 2009 | ★ | ★ | ★ | ★ | ★★ | ★ |  | ★ | 8 |
| Jeffrey M. Venstrom 2003 | ★ | ★ | ★ | ★ | ★ | ★ |  |  | 6 |
| Jens Gunther Brockmann 2018 | ★ | ★ | ★ | ★ | ★★ | ★ |  | ★ | 8 |
| Jonathan A. Fridell 2018 | ★ | ★ | ★ | ★ | ★ | ★ | ★ | ★ | 8 |
| Muthusamy 2008 | ★ | ★ | ★ | ★ | ★★ | ★ |  |  | 7 |
| M.R. Laftavi 2014 | ★ | ★ | ★ | ★ | ★ | ★ | ★ | ★ | 8 |
| M. Durlik 2018 | ★ | ★ | ★ | ★ | ★ | ★ | ★ | ★ | 8 |
| Nedo Poommipanit 2010 | ★ | ★ | ★ | ★ | ★ | ★ | ★ | ★ | 8 |
| Perosa M. 2011 | ★ | ★ | ★ | ★ | ★ | ★ | ★ |  | 7 |
| Pedro Ventura-Aguiar 2018 | ★ | ★ | ★ | ★ | ★★ | ★ | ★ | ★ | 9 |
| Rainer WG 2004 | ★ | ★ | ★ | ★ | ★ | ★ |  |  | 6 |
| Robert Ollinger 2012 | ★ | ★ | ★ | ★ | ★ | ★ | ★ |  | 7 |
| R.J. Stratta 2014 | ★ | ★ | ★ | ★ | ★★ | ★ | ★ | ★ | 9 |
| Sandesh Parajuli 2019 | ★ | ★ | ★ | ★ | ★★ | ★ | ★ |  | 8 |
| Timothy S. Larson 2004 | ★ | ★ | ★ | ★ | ★★ | ★ | ★ |  | 8 |
| Tadahiiro Uemura 2011 | ★ | ★ | ★ | ★ | ★★ | ★ |  | ★ | 8 |
| T. Ito 2018 | ★ | ★ |  | ★ | ★ | ★ | ★ |  | 6 |
| Tomimaru 2021 | ★ | ★ | ★ | ★ | ★ | ★ | ★ |  | 7 |

Note: Newcastle-Ottawa Scale contains 8 items within 3 domain and the total maximum score is 9. A study with score from 7-9, has high quality, 4-6, high risk, and 0-3 very high risk of bias. A study can be awarded a maximum of one star for each numbered item within the Selection and Outcome categories. A maximum of two stars can be given for Comparability.

**Table S3.**

| **Table S3. Rejection complication reported in included studies** | | | | | | |
| --- | --- | --- | --- | --- | --- | --- |
| First author and year | Pancreas | | | Kidney | | |
|  | Rejection Definition | Rate (%): PAK vs SPK | Treatment | Rejection Definition | Rate (%): PAK VS SPK | Treatment |
| Abhinav Humar 2001 | A decrease in urinary amylase  levels of 25% or more from baseline on 2 consecutive measurements or by a rise in the serum amylase or lipase  levels. Confirmed by  percutaneous biopsy | 9.3 vs 3.6 | A course of antilymphocyte therapy | Kidney graft biopsy | 0.5 vs 2.6 | A course of antilymphocyte therapy |
| Ayhan Dinckan 2012 | Pancreas graft biopsy | 17.6 vs 23.8 | A 1 gr/day pulse steroid treat­ment for 3 days, as the other patients received ATG (2.5 mg/kg/day). | Kidney graft biopsy | 17.6 vs 23.8 | Pulse steroid treatment/ treated with ATG |
| Bor-Uei Shyr 2021 | Pancreas graft biopsy | 16.7 vs 29.7 | ATG, 1 mg/kg daily from postoperative days 1 to 7 | NA | NA | NA |
| Fridell JA 2009 | Pancreas graft biopsy | 3.3 vs 0.7 | Not mention | NA | NA | NA |
| Jens Gunther Brockmann 2018 | C4d staining + Pancreas transplant biopsy | 88.2 vs 0.3 | A pulse of steroids+ lymphocyte depleting agent (ATG) | NA | NA | NA |
| Muthusamy 2008 | Confirmed retrospectively by a positive response to increased immunosuppression | 26.7 vs 21.7 | Three doses of methylprednisolone  (Solu-medrol) along with an increase in baseline immunosuppression-  either increasing the dose of tacrolimus or myphenolate or the introduction  of steroids | NA | NA | NA |
| Nedo Poommipanit 2010 | Pancreas graft biopsy | 6.9 vs 2.6 | Not mention | Kidney graft biopsy | 5.2 vs 5.9 | Not mention |
| Pedro Ventura-Aguiar 2018 | Based on clinical  criteria and pancreas graft biopsy | 66.7 vs 31.7 | Grade I was treated with methylprednisolone 500mg for 3 consecutive days, and grade II–III treated additionally with T-cell-depleting antibodies for 7 consecutive days | NA | NA | NA |
| Robert Ollinger2012 | Pancreas graft biopsy | 23.4 vs 16.7 | ATG induction | NA | NA | NA |
| R.J. Stratta 2014 | An unexplained rise in serum amylase, lipase, or  glucose levels and confirmed by ultrasound-guided percutaneous  pancreas biopsy | 22.9 vs 13.0 | Intravenous steroids and prompted a follow-up biopsy | An unexplained rise in serum creatinine level of >0.3 mg/dL or a 25% increase from baseline level and confirmed by ultrasound-guided percutaneous kidney biopsy | 28.6 vs 29.0 | Intravenous steroids and prompted a follow-up biopsy. |
| Sandesh Parajuli 2019 | Graded by Banff criteria scoring of pancreas graft biopsy | 37.5 vs 16.0 | IV steroid pulse with or  without ATG 6‐12 mg/kg in 4‐10 divided  doses | Kidney graft biopsy | 16.7 vs 23.4 | Steroid pulse plus ATG, 6‐10.5 mg/kg in 4‐7 divided doses |
| Timothy S. Larson 2004 | Pancreas graft biopsy | 4.3 vs 4.0 | OKT3 (5 mg/day for 10 days) + prednisone | NA | NA | NA |
| Tadahiiro Uemura 2011 | Pancreas graft biopsy | 20.0 vs 47.1 | Antibody treatment | NA | NA | NA |
| Tomimaru 2021 | Pancreas graft biopsy | 19.7 vs 1.7 | Not mention | NA | NA | NA |

Abbreviation: ATG, Anti-thymocyte globulin; OKT3, OKT3 Monoclonal Antibody; NA, Not Applicable.

**References**

1. Humar A, Ramcharan T, Kandaswamy R, Matas A, Gruessner RW, Gruessner AC et al. Pancreas after kidney transplants. Am J Surg. 2001 Aug;182(2):155-161. doi: 10.1016/s0002-9610(01)00676-6. PMID: 11574088.
2. Dinckan A, Aliosmanoglu I, Kocak H, Sari R, Erdogan O, Ertug Z et al. Pancreas survival in simultaneous pancreas-kidney and pancreas-after-kidney transplantations: a five-year follow-up report. Ann Transplant. 2012 Jul-Sep;17(3):14-19. doi: 10.12659/aot.883453. PMID: 23018251.
3. Kasiske BL, Gustafson S, Salkowski N, Stock PG, Axelrod DA, Kandaswamy R et al. Optimizing the program-specific reporting of pancreas transplant outcomes. Am J Transplant. 2013 Feb;13(2):337-347. doi: 10.1111/ajt.12036. Epub 2013 Jan 7. PMID: 23289524.
4. Shyr BU, Shyr BS, Chen SC, Shyr YM, Wang SE. Inferior survival outcomes of pancreas transplant alone in uremic patients. Sci Rep. 2021 Oct 26;11(1):21073. doi: 10.1038/s41598-021-00621-y. PMID: 34702876; PMCID: PMC8548435.
5. Huang E, Wiseman A, Okumura S, Kuo HT, Bunnapradist S. Outcomes of preemptive kidney with or without subsequent pancreas transplant compared with preemptive simultaneous pancreas/kidney transplantation. Transplantation. 2011 Nov 27;92(10):1115-1122. doi: 10.1097/TP.0b013e31823328a6. PMID: 21959215.
6. Farney AC, Cho E, Schweitzer EJ, Dunkin B, Philosophe B, Colonna J et al. Simultaneous cadaver pancreas living-donor kidney transplantation: a new approach for the type 1 diabetic uremic patient. Ann Surg. 2000 Nov;232(5):696-703. doi: 10.1097/00000658-200011000-00012. PMID: 11066142; PMCID: PMC1421224.
7. Fridell JA, Mangus RS, Hollinger EF, Taber TE, Goble ML, Mohler E et al. The case for pancreas after kidney transplantation. Clin Transplant. 2009 Aug-Sep;23(4):447-453. doi: 10.1111/j.1399-0012.2009.00996.x. Epub 2009 May 13. PMID: 19453642.
8. Venstrom JM, McBride MA, Rother KI, Hirshberg B, Orchard TJ, Harlan DM. Survival after pancreas transplantation in patients with diabetes and preserved kidney function. JAMA. 2003 Dec 3;290(21):2817-2823. doi: 10.1001/jama.290.21.2817. Erratum in: JAMA. 2004 Apr 7;291(13):1566. PMID: 14657065.
9. Gunther Brockmann J, Butt A, AlHussaini HF, AlMana H, AlSaad K, Al-Awwami M et al. Protocol Duodenal Graft Biopsies Aid Pancreas Graft Surveillance. Transplantation. 2019 Mar;103(3):622-629. doi: 10.1097/TP.0000000000002412. PMID: 30113993.
10. Fridell JA, Niederhaus S, Curry M, Urban R, Fox A, Odorico J. The survival advantage of pancreas after kidney transplant. Am J Transplant. 2019 Mar;19(3):823-830. doi: 10.1111/ajt.15106. Epub 2018 Oct 4. PMID: 30188614.
11. Muthusamy AS, Vaidya AC, Sinha S, Roy D, Elker DE, Friend PJ. Alemtuzumab induction and steroid-free maintenance immunosuppression in pancreas transplantation. Am J Transplant. 2008 Oct;8(10):2126-2131. doi: 10.1111/j.1600-6143.2008.02373.x. PMID: 18828772.
12. Laftavi MR, Pankewycz O, Gruessner A, Brian M, Kohli R, Feng L et al. Long-term outcomes of pancreas after kidney transplantation in small centers: is it justified? Transplant Proc. 2014 Jul-Aug;46(6):1920-1923. doi: 10.1016/j.transproceed.2014.06.044. PMID: 25131071.
13. Durlik M, Baumgart-Gryn K. Almost 200 Pancreas Transplantations: A Single-Center Experience. Transplant Proc. 2018 Sep;50(7):2124-2127. doi: 10.1016/j.transproceed.2018.02.097. Epub 2018 Mar 13. PMID: 30177122.
14. Poommipanit N, Sampaio MS, Cho Y, Young B, Shah T, Pham PT et al. Pancreas after living donor kidney versus simultaneous pancreas-kidney transplant: an analysis of the organ procurement transplant network/united network of organ sharing database. Transplantation. 2010 Jun 27;89(12):1496-1503. doi: 10.1097/TP.0b013e3181dd3587. PMID: 20414150.
15. Perosa M, Crescentini F, Noujaim H, Mota LT, Branez JR, Ianhez LE et al. Over 500 pancreas transplants by a single team in São Paulo, Brazil. Clin Transplant. 2011 Jul-Aug;25(4):E422-429. doi: 10.1111/j.1399-0012.2011.01470.x. Epub 2011 May 9. PMID: 21554397.
16. Ventura-Aguiar P, Ferrer J, Revuelta I, Paredes D, de Sousa-Amorim E, Rovira J et al. Pancreas outcomes between living and deceased kidney donor in pancreas after kidney transplantation patients. Nephrol Dial Transplant. 2018 Nov 1;33(11):2052-2059. doi: 10.1093/ndt/gfy133. PMID: 29893956.
17. Gruessner RW, Sutherland DE, Gruessner AC. Mortality assessment for pancreas transplants. Am J Transplant. 2004 Dec;4(12):2018-2026. doi: 10.1111/j.1600-6143.2004.00667.x. PMID: 15575904.
18. Ollinger R, Margreiter C, Bösmüller C, Weissenbacher A, Frank F, Schneeberger S et al. Evolution of pancreas transplantation: long-term results and perspectives from a high-volume center. Ann Surg. 2012 Nov;256(5):780-786; discussion 786-787. doi: 10.1097/SLA.0b013e31827381a8. Erratum in: Ann Surg. 2013 Mar;257(3):570. PMID: 23095622.
19. Stratta RJ, Farney AC, Orlando G, Farooq U, Al-Shraideh Y, Rogers J. Similar results with solitary pancreas transplantation compared with simultaneous pancreas-kidney transplantation in the new millennium. Transplant Proc. 2014 Jul-Aug;46(6):1924-1927. doi: 10.1016/j.transproceed.2014.05.079. PMID: 25131072.
20. Parajuli S, Arunachalam A, Swanson KJ, Aziz F, Garg N, Redfield RR et al. Outcomes after simultaneous kidney-pancreas versus pancreas after kidney transplantation in the current era. Clin Transplant. 2019 Dec;33(12):e13732. doi: 10.1111/ctr.13732. Epub 2019 Nov 6. PMID: 31628870.
21. Larson TS, Bohorquez H, Rea DJ, Nyberg SL, Prieto M, Sterioff S et al. Pancreas-after-kidney transplantation: an increasingly attractive alternative to simultaneous pancreas-kidney transplantation. Transplantation. 2004 Mar 27;77(6):838-843. doi: 10.1097/01.tp.0000114611.73689.3b. PMID: 15077023.
22. Uemura T, Ramprasad V, Matsushima K, Shike H, Valania T, Kwon O et al. Single dose of alemtuzumab induction with steroid-free maintenance immunosuppression in pancreas transplantation. Transplantation. 2011 Sep 27;92(6):678-685. doi: 10.1097/TP.0b013e31822b58be. PMID: 21841541; PMCID: PMC3353259.
23. Ito T, Kenmochi T, Aida N, Kurihara K, Kawai A, Ito T. Effectiveness of Preceding Solo Kidney Transplantation for Type 1 Diabetes With End-Stage Renal Failure. Transplant Proc. 2018 Dec;50(10):3249-3254. doi: 10.1016/j.transproceed.2018.06.014. Epub 2018 Jun 22. PMID: 30577193.
24. Tomimaru Y, Eguchi H, Doki Y, Ito T, Kenmochi T. Current state of pancreas transplantation in Japan based on the nationwide registry. Ann Gastroenterol Surg. 2021 Jan 25;5(4):494-501. doi: 10.1002/ags3.12423. PMID: 34337298; PMCID: PMC8316743.
